# Une tumeur hépatique rare chez l'enfant: l'hyperplasie nodulaire focale

**DOI:** 10.11604/pamj.2014.17.25.2645

**Published:** 2014-01-17

**Authors:** Mohamed Rami, Youssef Bouabdallah

**Affiliations:** 1 Service de Chirurgie Pédiatrique, CHU Hassan II, Fès, Maroc

**Keywords:** Tumeur hépatique, hyperplasie nodulaire, hépatoblastome, hepatic tumor, nodular hyperplasia, hepatoblastoma

## Image en medicine

Les tumeurs hépatiques de l'enfant sont rares, elles représentent 1 à 4% des tumeurs solides. L'hépatoblastome est le chef de fil des masses hépatiques. Nous rapportons le cas d'un garçon de 4 ans, déjà suivi pour une hémihypertrophie corporelle gauche, et dont le suivi en consultation a permis de diagnostiquer une masse du foie gauche. L'examen trouvait une voussure épigastrique d'environ 10 cm de diamètre, et une hémihypertrophie corporelle gauche. L'imagerie a objectivé une masse de 9 cm aux dépens du foie gauche, tissulaire, vascularisée. Le patient fut opérer, on a découvert une masse ferme, avec une artère hépatique gauche distincte. Les suites opératoires étaient simples. L’étude anatomopathologique était en faveur d'une hyperplasie nodulaire focale, qui est très rare chez l'enfant (11% des tumeurs hépatiques, et 0,45% des tumeurs).

**Figure 1:**
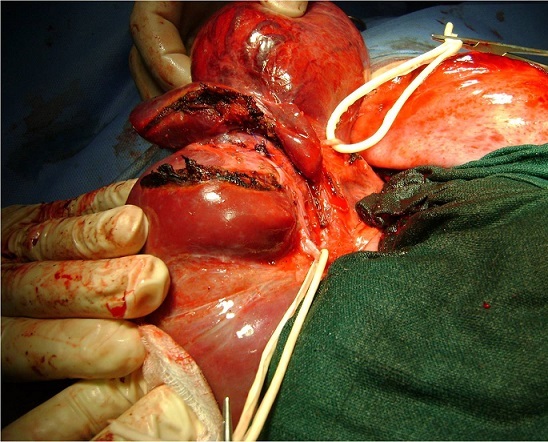
Image per opératoire montrant une tumeur du foie gauche avec une artère hépatique gauche distincte

